# Exogenous enzymes and probiotics alter digestion kinetics, volatile fatty acid content and microbial interactions in the gut of Nile tilapia

**DOI:** 10.1038/s41598-021-87408-3

**Published:** 2021-04-15

**Authors:** Roel M. Maas, Yale Deng, Yueming Dersjant-Li, Jules Petit, Marc C. J. Verdegem, Johan W. Schrama, Fotini Kokou

**Affiliations:** 1 Aquaculture and Fisheries Group, Wageningen University and Research, Wageningen, The Netherlands; 2 Danisco Animal Nutrition, Oegstgeest, The Netherlands

**Keywords:** Animal physiology, Dietary carbohydrates, Ichthyology, Microbiome, Gastrointestinal system

## Abstract

Sustainable aquafeed production requires fishmeal replacement, leading to an increasing use of plant-derived ingredients. As a consequence, higher levels of antinutritional substances, such as non-starch polysaccharides and phytate, are present in aquafeeds, with negative effects on fish performance, nutrient digestibility and overall gut health. To alleviate these negative effects, providing exogenous digestive enzymes and/or probiotics can be an effective solution. In this study, we tested the effect of dietary supplementation of enzymes (phytase and xylanase) and probiotics (three strains of *Bacillus amyloliquefaciens*) on nutrient digestion kinetics and volatile fatty acid content along the gut, and the distal gut microbiome diversity in Nile tilapia. Chyme volatile fatty content was increased with probiotic supplementation in the proximal gut, while lactate content, measured for the first time in vivo in fish, decreased with enzymes along the gut. Enzyme supplementation enhanced crude protein, Ca and P digestibility in proximal and middle gut. Enzymes and probiotics supplementation enhanced microbial interactions as shown by network analysis, while increased the abundance of lactic acid bacteria and *Bacillus* species. Such results suggest that supplementation with exogenous enzymes and probiotics increases nutrient availability, while at the same time benefits gut health and contributes to a more stable microbiome environment.

## Introduction

Aquaculture is one of the fastest-growing food production sectors providing more than half of the fish supply around the world^1^. To meet the increasing demand for feeds while ensuring a sustainable growth for aquaculture, there is a trend over the past decades to switch from fisheries-dependent fish meal as the main protein ingredient to widely-available plant-based ingredients^2^. However, commonly used plant-based ingredients such as soybean, rapeseed and sunflower meals, contain a wide variety of antinutritional factors that impair fish performance^3^. Non-starch polysaccharides (NSP), protease inhibitors and phytic acid (i.e., phytate) are antinutritional factors reported to negatively affect nutrient digestibility and mineral absorption (Fe, Mg, Zn, Cu, Ca and P), reducing the nutrient utilization efficiency and fish growth^3,4^. Consequently, to be able to efficiently use plant-based ingredients in the fish feeds, it is important to alleviate their antinutritional effects. For instance, phytate bound P generally remains unavailable for fish, because of the lack of endogenous enzymes to break down phytate. Inorganic P needs to be supplemented in aquafeeds that contain high levels of plant-based ingredients in order to fulfil the P requirements; this is undesired as mined P is exhaustible. Improving the bioavailability with phytate bound P with phytase, reduces the dependency of aquaculture on inorganic P sources^5,6^.

As monogastric animals, fish lack NSP-degrading enzymes such as β-glucanases and β-xylanases, that allow digestion of the long polysaccharide chains present in NSP-rich plant-based ingredients, and therefore lack the ability to efficiently utilize nutrients from plant-based diets^7^. Commercially, to increase the nutrient availability and digestibility of the feeds, exogenous enzymes, such as phytase and xylanase, are commonly applied in the animal feed formulations, that catalyze the hydrolysis of phytic acid and polymeric carbohydrates, respectively^8^. Addition of phytase and β-xylanase was reported to improve nutrient digestibility and growth in several fish species^9–13^. The digestion of NSP in the gastrointestinal tract (GIT) is associated with fermentation by commensal microbes, producing beneficial volatile fatty acids (VFA), which are rapidly absorbed by the intestine^4,14^. The type of carbohydrates in the diet can have significant effects on the composition of the intestinal microbiota and consequently on the type and amount of VFAs produced^15^. The principle VFAs produced in the GIT of monogastric terrestrial animals are acetate, propionate and butyrate, and at low concentrations also formate, valerate, caproate, isobutyrate and isovalerate^16^. In the fish GIT, the principle VFA produced is acetate, while propionate and butyrate are produced at lower levels^7^. Levels of lactic acid, which is commonly found in the GIT of monogastric animals like pigs^17^, have not been reported in fish, although an in vitro study by Leenhouwers et al^18^ showed a potential of lactic acid production by inoculum collected from the GIT of Nile tilapia (*Oreochromis niloticus*).

Dietary supplementation with probiotics is reported to have positive effects on fish health and disease resistance. Recent studies also showed the dietary probiotics can enhance the nutrient digestibility of aquatic animals by increasing digestive enzymes activity^19^. Among the probiotic candidates, *Bacillus* species have been widely used in aquaculture, due to their sporulation capacity and positive effects on feed utilization, immune response^20^ and digestive enzyme activity such as protease, amylase, trypsin, and lipase^21,22^. Besides, supplementation of some *Bacillus* species through water bath or live feed was shown to modulate the gut microbial composition in fish^23,24^. Studies in aquatic and livestock animals using next generation sequencing technology to map the gut microbiome reported that probiotics may affect the microbiome structure by steering microbial interactions, potentially promoting intestinal homeostasis^25,26^. Several studies in white leg shrimp, gilthead sea bream and Nile tilapia revealed that *Bacillus* species could change the bacterial diversity and proportional composition of bacterial phyla in the gut^27,28^. However, it remains unclear how *Bacillus* species improve the utilization efficiency of plant-based diets and how they may influence microbial structure in the fish GIT.

Nile tilapia is the third most important aquaculture species by volume^1^, with omnivorous dietary habits and the potential for GIT fermentation. Supplementing plant-based diets with phytase and β-xylanases improved growth and digestibility of dry matter, crude protein, carbohydrate and ash, showing a good potential for application in Nile tilapia^11,12^. Several studies have been performed so far using *Bacillus* strains as probiotics in Nile tilapia diets, however, focusing mainly on the effects on immune response and growth performance^29–31^. To our knowledge, no studies so far have focused on either the impact of exogenous enzymes or probiotics and their combination on the kinetics of macro- and micronutrient digestion and VFA production along the GIT of Nile tilapia. The combined supplementation of exogenous enzymes and probiotic could result in a complimentary mode of action. The ability to produce digestive enzymes (including NSP-degrading enzymes) by the probiotic may complement the endogenous enzyme activity. Moreover, the exogenous enzymes may increase the availability of suitable substrate for the probiotic as well as may promote the growth of beneficial bacteria in the GIT. The aim of this study was to improve the nutritional utilization efficiency of plant-based diets for Nile tilapia by increasing NSP digestibility and digestive enzyme activity or the gut microbiome structure. For this purpose, we assessed the effect of dietary supplementation of an enzymatic cocktail (phytase and β-xylanase) and a cocktail of three probiotic *Bacillus* strains on (1) the kinetics of the digestion of dry matter, crude protein and minerals of the diet; (2) the VFA and lactate concentrations along the GIT; and (3) the gut microbiome structure of Nile tilapia. This was experimentally tested in a two-by-two factorial design to quantify the effects of enzyme and probiotic addition (factors), and their interactions.

## Results

### VFA and lactic acid content along the gastrointestinal tract

**Figure 1:**
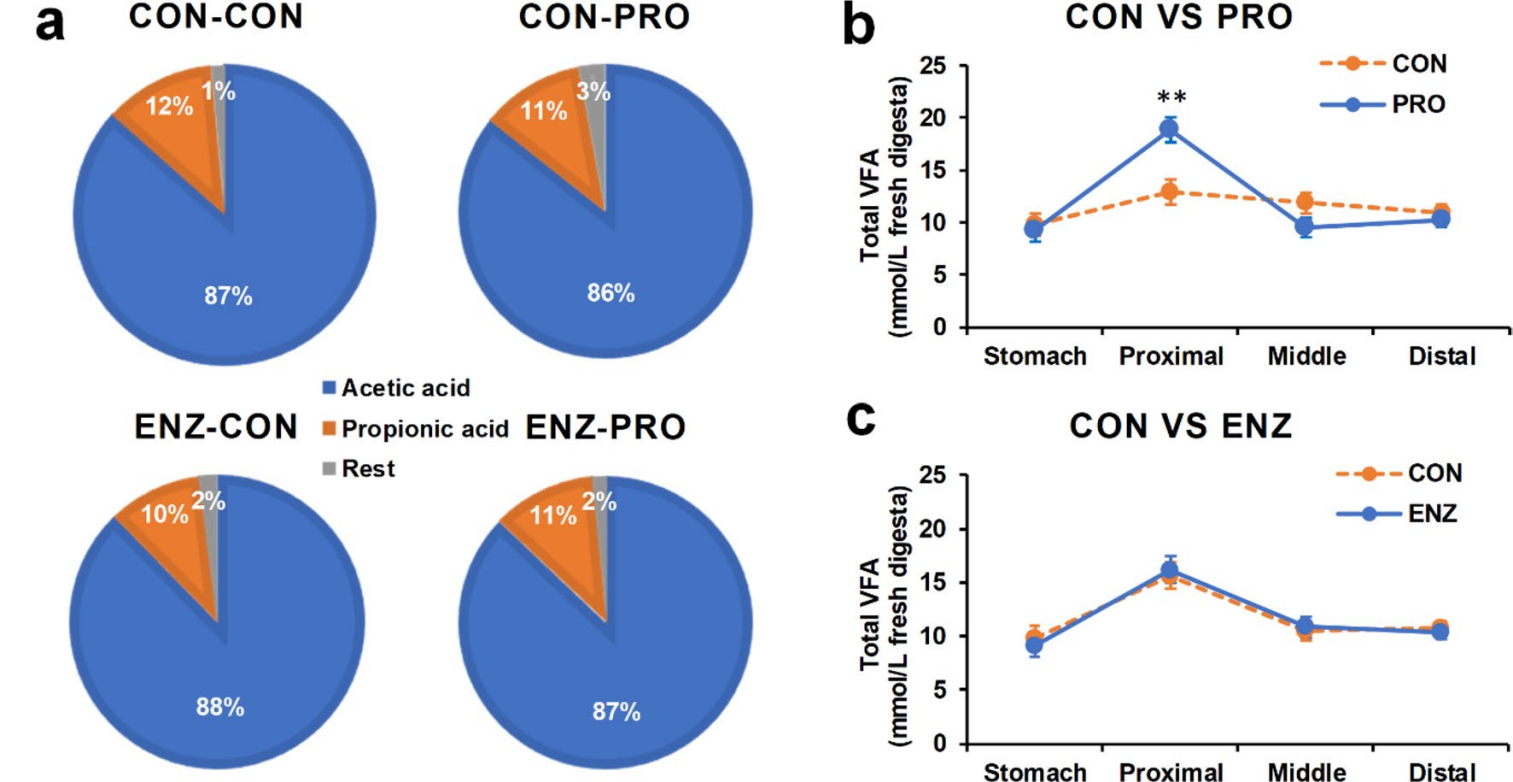
Volatile fatty acid (VFA) composition in fresh chyme along the gastrointestinal tract of Nile tilapia. (**a**) The proportion of the two most abundant VFAs per dietary treatment averaged over the four segments of the gastrointestinal tract. (**b**) The main effect of probiotics supplementation on the total VFA content in fresh chyme along the gastrointestinal tract. (**c**) The main effect of enzyme supplementation on the total VFA content in fresh chyme along the gastrointestinal tract. Error bars indicate standard error of means; **P < 0.01. CON-CON, no enzymes or probiotics added; CON-PRO, probiotics added; ENZ-CON, enzymes added; ENZ-PRO, enzymes and probiotics added.

The chyme VFA composition along the GIT of fish fed with the four different diets was measured (Fig. 1), showing a dominance of acetic acid (86–88%), followed by propionic acid (10–12%) (Fig. 1a). (Iso) butyric and (iso) valeric acid acids were also detected in lower concentrations (in total 1–3%)(Table 1). Our measurements indicated that overall the highest total VFA concentration was in the proximal gut, while the stomach, middle and distal intestine had a similar total VFA content. Although there were no significant differences on the VFA composition of the different dietary treatments, probiotics supplementation (CON-PRO) was observed to increase the total VFA content in the proximal gut compared to the control diet (CON-CON; *P* = 0.005; Fig. 1b).

**Table 1:** The volatile fatty acids (VFA) content along the gastrointestinal tract of Nile tilapia fed with four experimental diets.

Enzymes	CON		ENZ		SEM	*P* value		
Probiotics	CON	PRO	CON	PRO		ENZ	PRO	ENZ*PRO
**Volatile fatty acids (mM/L fresh digesta)**								
Stomach								
Acetic acid	7.2	8.8	8.5	6.1	1.3	ns	ns	*
Propionic acid	1.6	1.3	1.2	1.4	0.3	ns	ns	ns
Iso butyric acid	nd	< 0.1	0.1	nd	na	na	na	na
Butyric acid	0.2	0.4	0.3	0.2	na	na	na	na
Iso valeric acid	0.1	< 0.1	0.1	< 0.1	na	na	na	na
Valeric acid	0.2	< 0.1	0.2	0.1	na	na	na	na
Total	**9.2**	**10.6**	**10.5**	**7.8**	1.5	ns	ns	ns
Proximal								
Acetic acid	9.7	16.2	12.2	15.2	1.6	ns	**	ns
Propionic acid	1.8	3.0	2.1	2.4	0.4	ns	#	ns
Iso butyric acid	nd	nd	nd	nd	na	na	na	na
Butyric acid	nd	0.5	nd	0.2	na	na	na	na
Iso valeric acid	< 0.1	0.1	< 0.1	< 0.1	na	na	na	na
Valeric acid	< 0.1	0.1	< 0.1	< 0.1	na	na	na	na
Total	**11.5**	**19.8**	**14.4**	**18.0**	1.7	ns	**	ns
Middle								
Acetic acid	10.5	8.5	11.3	9.0	1.2	ns	ns	ns
Propionic acid	1.0	0.7	0.7	0.6	0.3	ns	ns	ns
Iso butyric acid	nd	< 0.1	nd	nd	na	na	na	na
Butyric acid	0.1	0.2	nd	0.1	na	na	na	na
Iso valeric acid	0.1	< 0.1	< 0.1	nd	na	na	na	na
Valeric acid	0.1	0.1	< 0.1	nd	na	na	na	na
Total	**11.7**	**9.4**	**12.0**	**9.7**	1.3	ns	ns	ns
Distal								
Acetic acid	10.1	9.7	10.1	9.0	0.9	ns	ns	ns
Propionic acid	0.8	0.8	1.0	0.7	0.2	ns	ns	ns
Iso butyric acid	nd	nd	nd	nd	na	na	na	na
Butyric acid	0.1	0.1	nd	nd	na	na	na	na
Iso valeric acid	nd	nd	nd	nd	na	na	na	na
Valeric acid	nd	nd	nd	nd	na	na	na	na
Total	**10.9**	**10.6**	**11.0**	**9.7**	0.9	ns	ns	na

The total VFA content within each part of the GIT is indicated in bold.

CON; no supplementation; ENZ, enzyme (effect) supplementation; PRO, probiotic (effect) supplementation; ENZ*PRO, interaction effect; SEM, standard error of means; nd, not detectable, na, not applicable, ns, not significant, # *P* < 0.1, *P < 0.05, **P < 0.01.

**Figure 2:**
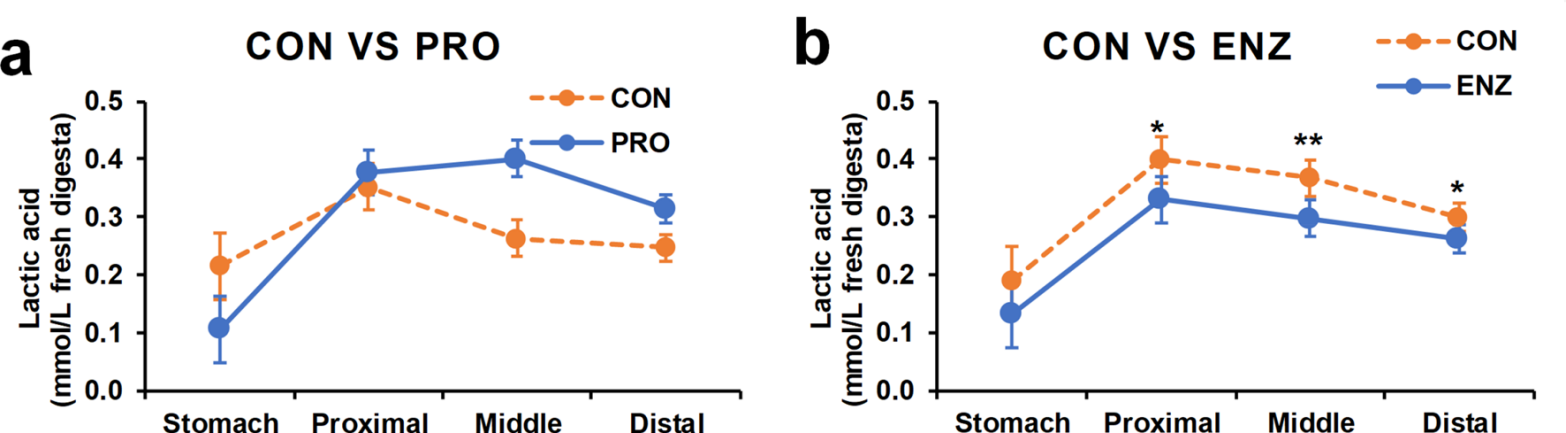
The lactic acid concentration in fresh chyme along the gastrointestinal tract of Nile tilapia. (**a**) The main effect of probiotics supplementation on the lactic acid concentration. (**b**) The main effect of enzyme supplementation on the lactic acid concentration. Error bars indicate standard error of means; *P < 0.05; **P < 0.01. CON-PRO, probiotics added; ENZ-CON, enzymes added; ENZ-PRO, enzymes and probiotics added.

Lactic acid was also detected in the chyme of Nile tilapia GIT, ranging from 0.08 mM in the stomach to 0.43 mM in the proximal gut and decreasing further in the distal gut (Fig. 2; Table 2). Interestingly, we observed that enzyme supplementation reduced lactic acid concentration in the proximal, middle and distal gut (*P* < 0.05; Fig. 2b). A different trend was observed by the probiotics showing an increase in the lactic acid concentration after the stomach; however, we did not find a significant difference with the control (*P* > 0.1).

**Table 2:** The lactic acid content in fresh chyme along the gastrointestinal tract of Nile tilapia.

Enzymes	CON		ENZ		SEM	*P* value		
Probiotics	CON	PRO	CON	PRO		ENZ	PRO	ENZ*PRO
**Lactate (mmol/L fresh digesta)**								
Stomach	0.25	0.08	0.28	0.18	0.08	ns	ns	ns
Proximal	0.43	0.38	0.25	0.28	0.06	*	ns	ns
Middle	0.33	0.39	0.24	0.20	0.04	**	ns	ns
Distal	0.29	0.32	0.20	0.20	0.03	*	ns	ns

CON, no supplementation; ENZ, enzyme (effect) supplementation; PRO, probiotic (effect) supplementation; ENZ*PRO, interaction effect; ns, not significant, *P < 0.05, **P < 0.01.

### Minerals absorption and digestibility along the gastrointestinal tract

**Figure 3:**
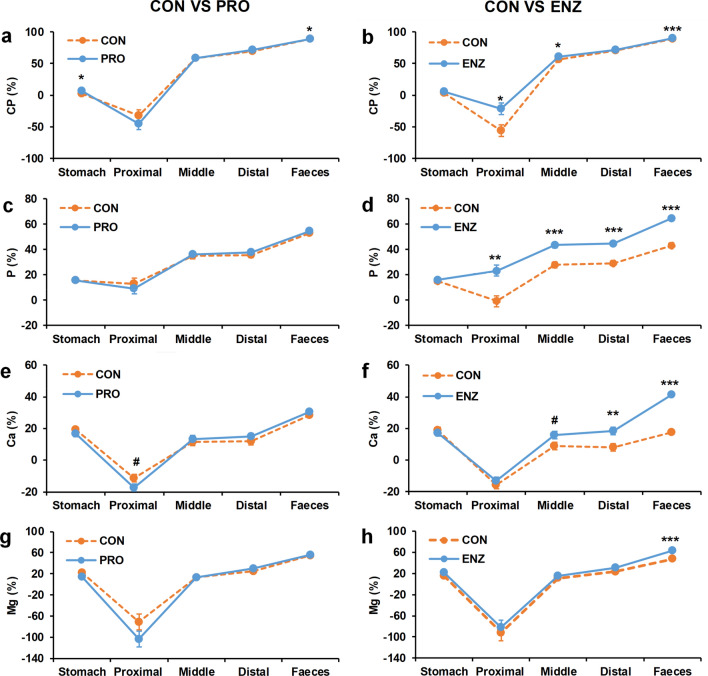
The main effect of probiotic and enzyme supplementation on the kinetics of apparent digestibility coefficient (ADC) in Nile tilapia GIT. The ADC values represent the cumulative ADC from stomach to faeces passing through the proximal, middle and distal gut. The ADC of: crude protein (CP) (panel **a**,**b**); phosphorus (P) (panel **c**,**d**); calcium (Ca) (panel **e**,**f**); and magnesium (Mg) (panel **g**,**h**). In panel a, c, e and g (left side) the main effect of dietary probiotics mixture supplementation on ADC values is shown and in panel b, d, f and h (right side) the main effect of dietary enzyme supplementation on ADC values is shown. The ADC in the stomach, proximal, middle and distal were determined on collected digesta in current study. The faecal ADCs were determined on faeces collected by settling columns during the growth trail (published in^57^). Error bars indicate standard error of means. CON-PRO, probiotics added; ENZ-CON, enzymes added; ENZ-PRO, enzymes and probiotics added. Symbols: ns, not significant, #*P* < 0.1, *P < 0.05, **P < 0.01; *** P < 0.001.

Looking at the apparent digestibility coefficient (ADC) for crude protein (CP), phosphorus (P), calcium (Ca) and magnesium (Mg), we found large differences between the different parts of the GIT (Fig. 3; Table S1). For most nutrients, digestion started in the stomach as indicated by the small positive ADC values, but the largest part of digestion took place in the mid intestine. For CP and most minerals, the ADC in the proximal intestine was lower than the ADC in the stomach. For CP, Ca and Mg negative ADC values were even observed in the proximal intestine, most likely related to endogenous minerals and enzyme secretion in this gut segment (Fig. 3).

In relation to the treatments, enzyme supplementation increased the ADC of CP in the proximal and middle intestine (*P* < 0.05), while this effect was absent in the distal intestine (Table 3). Both enzyme and probiotic supplementation had a main effect on the ADC of CP (*P* < 0.05; Fig. 3b). Probiotics supplementation reduced the ADC of CP (averaged over the control treatments) from 89.4% (CON-CON and ENZ-CON) to 89.2% (CON-PRO and ENZ-PRO); whereas enzyme supplementation enhanced the ADC of CP (averaged over enzyme treatments) from 89.0% (CON-CON and CON-PRO) to 89.5% (ENZ-CON and ENZ-PRO). The ADC of P was enhanced by enzyme supplementation from the proximal intestine onward (*P* < 0.01; Fig. 3d). We estimated the levels of phytate in the diets of the current study to be approximately 4.2 g/kg diet, out of the 10.5 g/kg of total P. In addition to the absorption of P, enzyme supplementation improved Ca absorption, but this effect was more obvious in the distal part of the GIT (Fig. 3f; Table 3). The absorption of Mg was numerically higher in diets supplemented with enzymes, but this effect was only significant for faecal ADC of Mg (*P* < 0.05; Fig. 3g). The values for the nutrient/mineral availability in the stomach, proximal, middle and distal as well as the availability of the microminerals copper, iron, manganese and zinc are reported in Table 3 and Supplementary Table S1.

**Table 3:** The apparent digestibility coefficient (ADC %) in the distal gut of Nile tilapia, values for stomach, proximal and middle are given in Supplementary Table S1.

Enzymes	CON		ENZ		SEM	*P* values		
Probiotics	CON	PRO	CON	PRO		ENZ	PRO	ENZ*PRO
**ADC (%)**								
Dry matter	45.7	47.0	46.5	49.2	1.6	ns	ns	ns
Crude protein	69.7	70.4	69.7	72.7	1.5	ns	ns	ns
Ash	-6.8	-6.4	3.6	6.5	2.1	*	ns	ns
Phosphorous	29.2	28.9	42.0	46.8	1.2	***	ns	ns
Calcium	7.1	9.3	16.8	20.4	1.6	**	ns	ns
Magnesium	21.9	25.2	27.0	35.9	2.8	ns	ns	ns

CON; no supplementation; ENZ, enzyme (effect) supplementation; PRO, probiotic (effect) supplementation; ENZ*PRO, interaction effect; SEM, standard error of means; ns, not significant, *P < 0.05, **P < 0.01, ***P < 0.001.

### Microbial community structure and composition

**Figure 4:**
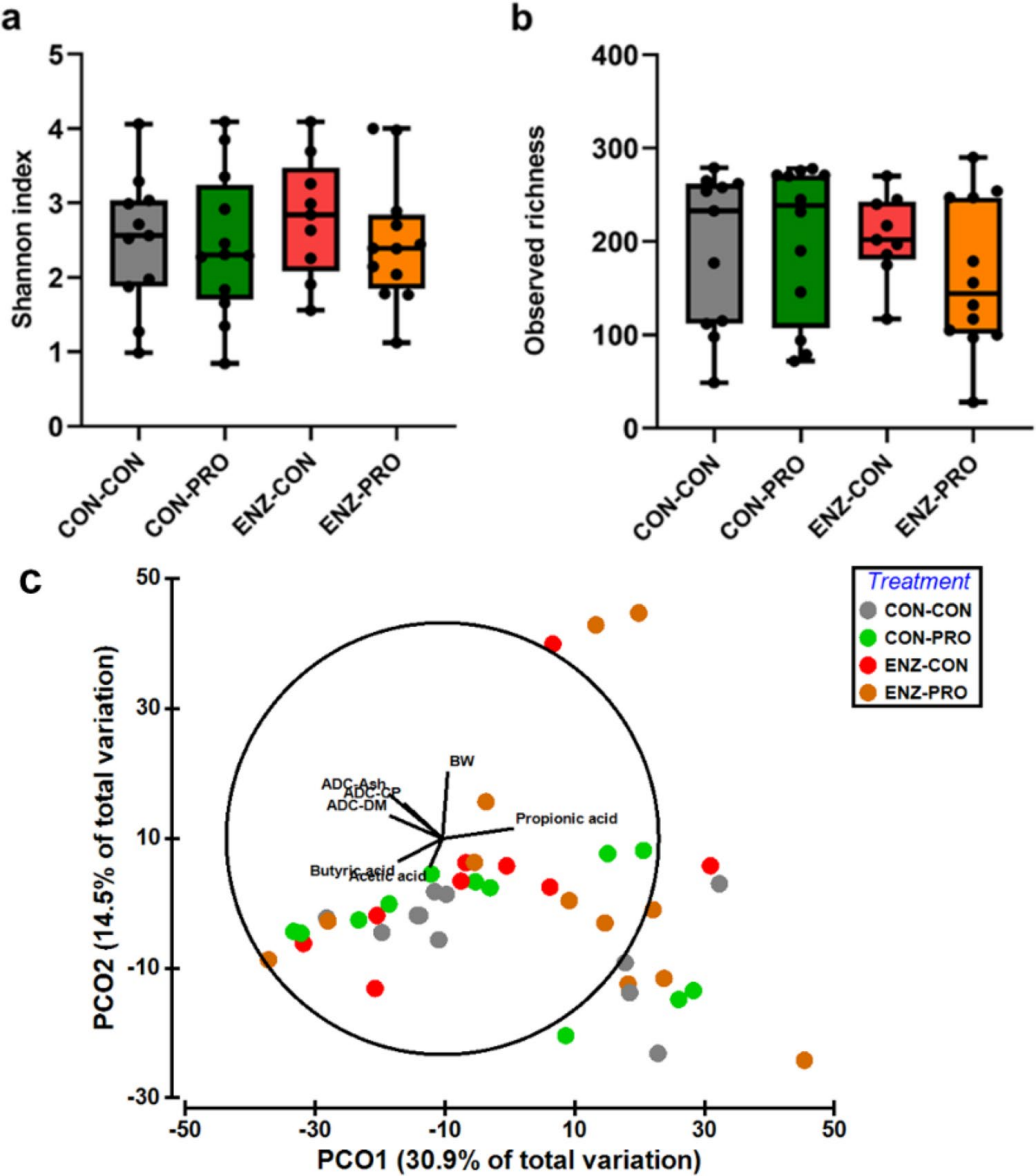
Microbial diversity in the distal gut of Nile tilapia. (**a**) Shannon index, (**b**) observed richness, and structure shown by (**c**) PCoA analysis based on Bray–Curtis distance and the correlation between microbial community structure and growth-related parameters. BW, body weight, ADC, apparent digestibility coefficient. Acetic acid, butyric acid and propionic acid are the concentrations from the distal gut. CON-CON, no enzymes or probiotics added; CON-PRO, probiotics added; ENZ-CON, enzymes added; ENZ-PRO, enzymes and probiotics added.

The microbial community structure and composition were evaluated in the distal gut of the fish fed the different dietary treatments, using 16S rRNA amplicon sequencing. When looking at the microbial community diversity and structure, no significance difference was found between the treatments, as indicated by the alpha-diversity—Shannon index and richness—and beta-diversity—PCoA and PERMANOVA analysis using Bray Curtis as a distance metric (Fig. 4). However, when the bacterial community composition (i.e. Operational Taxonomic Units table) in distal gut was further correlated with the final fish body weight (BW), VFA content and nutrient ADC in the distal gut, a weak but significant positive correlation was found between the propionic acid content and the gut microbiota samples along the PCo1 axis (mainly coming from ENZ-PRO diet; Fig. 4c; R^2^ = 0.136; *P* = 0.045).

**Figure 5:**
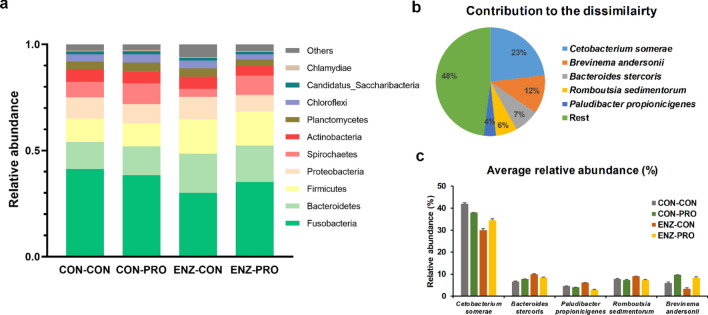
Microbial community composition, showing (**a**) the relative abundance of the most abundant phyla, (**b**) the top 5 species contributing the microbial composition dissimilarity among the dietary groups and (**c**) the relative abundance of the top 5 species. CON-CON (n = 11), no enzymes or probiotics added; CON-PRO (n = 12), probiotics added; ENZ-CON (n = 9), enzymes added; ENZ-PRO (n = 12), enzymes and probiotics added; error bars indicate standard error of means.

At the phylum level composition (Fig. 5a), Fusobacteria was the most abundant group (36.2%), followed by Bacteroidetes (15.5%) and Firmicutes (13.5%). Overall, 5 OTUs were found to contribute to 50% of the total dissimilarity between the treatments in microbial composition, as indicated by SIMPER analysis (Fig. 5b). *Cetobacterium somerae* was the most dominant species (36.1% of the total abundance) contributing to 23.0% of the dissimilarity among the dietary groups. The others four species, including *Brevinema andersonii*, *Bacteroides stercoris*, *Romboutsia sedimentorum* and *Paludibacter propionicigenes*, were also dominant species (27% of the total abundance), accounting for 28.9% of the dissimilarity among the dietary treatments. Enzyme supplementation increased the relative abundance of *B. stercoris*, *R. sedimentorum* and *P. propionicigenes* while the relative abundance of *B. andersonii* increased by probiotics. Enzyme and probiotic supplementation seem to have an overall positive effect on the lactic acid bacteria abundance (*Lactobacillales* order; Supplementary Figure S1) as well as in the abundance of OTUs belonging to the *Bacillus* genus (Supplementary Figure S2).

### Microbial co-occurrence network analysis

**Figure 6:**
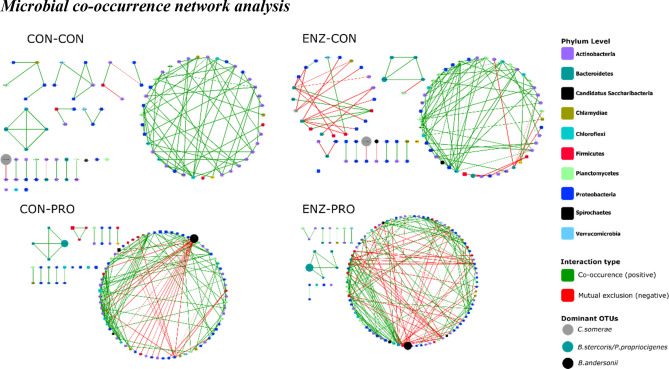
The microbial co-occurrence network in each of the four dietary groups. The nodes represent the interacting OTUs, coloured based on their phylum taxa. The size of each node is proportional to the relative abundance of the OTU. The edges are the lines connecting the OTUs, with colour indicating the type of interaction (green for copresence/positive co-occurrence; red for mutual exclusion/negative co-occurrence). The edge thickness corresponds to the statistical significance (the *P* value) of the correlation—the thicker the edge, the lower the *P* value. The dominant species were indicated in ellipse nodes. The networks were produced by CoNet app, within Cytoscape software (version 3.7.1; http://apps.cytoscape.org/apps/conet). CON-CON, no enzymes or probiotics added; CON-PRO, probiotics added; ENZ-CON, enzymes added; ENZ-PRO, enzymes and probiotics added.

A co-occurrence network analysis was performed to understand how microbial interactions may be affected by enzyme and probiotic dietary supplementation. Overall, after permutations and multiple correction tests, the co-occurrence network was mainly occupied by OTUs belonging to Proteobacteria, Bacteroidetes, Actinobacteria and Firmicutes phyla (Fig. 6), which are amongst the most abundant ones.

Regarding the network characteristics (Table 4), supplementation of probiotics or enzymes increased the total number of both nodes and edges in the network. A significant increase in the clustering co-efficiency and density of the network was observed by probiotics supplementation. Enzyme supplementation in the diets also increased the density of the network. The ratio of positive to negative edges was significantly reduced with enzyme and probiotic supplementation. These results indicate that mainly probiotics, and to some extent also enzyme supplementation, enhanced species-species (co-occurrence patterns) interactions, as well as the type of those interactions.

**Table 4:** The characteristics of the networks from each dietary group.

Enzymes	CON		ENZ	
Probiotics	CON	PRO	CON	PRO
Number of nodes	86	98	90	112
Number of edges	94	146	118	186
Number of positive edges	90	122	86	137
Number of negative edges	4	24	32	49
Positive/Negative ratio	23	5*	3*	3*
Clustering co-efficiency	0.19	0.22*	0.18	0.24*
Density	0.025	0.031*	0.029*	0.03*
Heterogeneity	0.77	0.98	0.86	0.96

CON, no supplementation; ENZ, enzyme supplementation; PRO, probiotic supplementation.

*Stars indicate significant differences from the CON-CON treatment at *P* < 0.05, after performing the permutations analysis (70% of the initial data).

## Discussion

### Volatile fatty acids and lactate content along the gastrointestinal tract

The digestion of NSP in tilapia is expected to be through anaerobic microbial glycolysis (fermentation), producing VFAs^7,32,33^. In the present study, we expected that the supplementation of an enzymatic cocktail containing xylanase (NSP-degrading enzyme) and phytase would improve the NSP digestibility, as previously shown in the Nile tilapia^34^. Such supplementation would stimulate the breakdown of the polysaccharides into readily available oligomers and monomers for fermentation, increasing the production of beneficial VFA and improving the nutrient utilization. The enzyme supplementation in the present study did increase the NSP digestibility by approximately 29% (data not shown,^57^). However, this increase did not go hand in hand with an increase in VFA levels, which remained unaffected by the enzyme supplementation (Fig. 1c). On the contrary, the supplementation of probiotics increased the level of acetic acid as well as the total VFA in the proximal part of the GIT (Fig. 1b). Similarly, a strain affiliated to *B.amyloliquefaciens* was reported to dramatically increase the concentration of acetate in the intestine of Nile tilapia, which could be explained by the enrichment of the short-chain fatty acid-producing bacteria^35^. Unfortunately, the microbial community in the proximal gut, where major changes were observed in our study, was not sampled and analysed. This should be considered for future research.

Overall, the total concentration of VFA along the GIT remained rather constant, with averages between 9.5 and 10.7 mmol/L fresh digesta. Generally, it is shown that the concentration of VFA increases towards the distal intestine, including two studies on Nile tilapia^36,37^. This coincides with increased microbial activity and anaerobic conditions in the distal gut^7,38^. The average concentration of total VFA found in the distal gut within the present study was 10.6 mmol/L fresh digesta, which is close to the average reported across fish species (with predominately herbivorous feeding habits), but considerably lower compared to the studies on Nile tilapia where on average 16.9 and 17.3 mmol VFA/L fresh digesta was found^36,37^. It could be speculated that the relative high levels of VFA found in the proximal gut of the intestine could have reduced the available substrate for fermentation in the distal part, thereby reducing the potential of VFA production. This is supported by the observation that the cumulative total VFA content of the different sections of the GIT (stomach + proximal + middle + distal) in this study, is highly comparable to the total VFA content in the GIT of Nile tilapia^36,37^. However, what caused this relative high levels of VFA observed in the proximal gut in this study remains unclear.

While several studies have investigated lactate in serum or under in vitro conditions in fish^39–41^, the present study is the first to measure the presence of lactate in the intestinal content of fish in vivo. Compared to the concentrations of the other VFAs, the observed level of lactate was very low. Nevertheless there was a clear trend visible in the concentrations over the different compartments of the GIT, i.e. low lactate concentrations in the stomach, relatively high in the proximal compartment and gradually decreasing towards the distal intestinal compartment. Interestingly, significant differences were observed among the dietary treatments. Supplementation with both xylanase and phytase resulted in lower lactate concentrations in each of the intestinal segments, except for the stomach. Possibly, the enzymatic supplementation has sped up the initial fermentation of oligosaccharides into acetic acid and lactic acid, resulting in earlier secondary fermentation of lactic acid into other VFAs, such as propionic acid and butyric acid^42^. Coinciding with the decrease in lactic acid, an increase in the presence of lactic acid bacteria was measured. While these bacteria predominantly produce lactic acid, it has been shown that lactic acid bacilli can also catabolize lactic acid into other compounds such as acetate^43^. This was further supported by an increase in species—species interaction that was observed following enzymatic supplementation.

In line with what is observed in other studies on fish, acetate (86–88%) is the dominant VFA found in the present study followed by propionate (10–12%) and the rest fraction (1–3%), of which butyrate forms the bulk^7^. In other monogastric animals, the order of prevalence of the different types of VFA is the same, although with a more proportional distribution; 60–75 acetate, 15–25% propionate and 10–15% butyrate^44^. In monogastric animals, VFA produced can be rapidly absorbed in the colonic lumen, with 95–99% of the VFA production being absorbed before reaching the rectum^45,46^. From studies in poultry and pigs, it is known that the route of uptake differs between types of VFA; acetate and propionate enter the blood passively, whereas butyrate is primarily used as direct source of energy by the colonocytes^16,45,47^. Likewise, we expect that in fish most of the VFA produced is rapidly absorbed, whereby it remains unclear whether the rate of uptake is different between acetate, propionate and butyrate. Therefore it is difficult to quantify the amount and composition of VFA produced in vivo based on measuring concentration in chyme, and such data should be interpreted with care.

### Nutrient digestibility and digestion kinetics along the gastrointestinal tract

Examining the digestibility coefficients along the GIT broadens insight in the digestion kinetics in fish, considering such studies in fish are rare. The digestion kinetics can enable us to pinpoint the locations in the GIT where probiotics and enzymes are active, or where minerals become available. One of the main goals of the present study was to increase P availability from dietary phytate by including exogenous enzymes in the diet, in order to reduce the dependency of finite P sources. In line with many studies using phytase, the enzyme supplementation improved the overall P availability^34,48–50^ throughout the GIT, starting from the proximal gut. Hereby the difference in P availability between enzyme supplementation (average ENZ-CON and ENZ-PRO) and no supplementation (average CON-CON and CON-PRO) in the proximal and complete gut was comparable with 20% and 23%, respectively. This indicates that the enzymes were active in the stomach and potentially in the proximal gut as enzymes supplementation did not further enhance the P availability. The activity of commercial phytase products is generally in a pH range of 2.5–5.5^49,51^. The low pH in the stomach helps to quickly break down phytate before it chelates with Ca^+2^ and other minerals. In tilapia, the pH in the stomach ranges from 1 to 4.5^37,52^, depending on the time post prandial feeding and the dietary composition^52^. From the proximal to the distal intestine, the pH is rather constant ranging between 6.4 and 7^37^. Such an increase in the pH after the stomach deactivates phytase activity in the GIT, therefore we expect phytase mainly to be active in the stomach. Although P is not absorbed in the stomach, in the current study we observed P availability between 13 and 18% (unaffected by treatment), while P availability is almost 0% in the proximal intestine when no enzyme was supplemented. It is known that low pH increases the solubility of several minerals^53^; such increased solubility can lead to a faster evacuation (liquid fraction) from the stomach to the proximal intestine, thus overestimating the availability of these minerals (including P, especially the monocalcium phosphate).

In this study, probiotics supplementation showed no effect on the digestion kinetics along the GIT, except a negative effect on Mn (Table S1). Certain strains that belong to *B. amyloliquefaciens* can synthesize many enzymes including amylase, cellulase, and xylanase, and therefore could potentially increase nutrient digestibility^54–56^. However, no beneficial effect of probiotics supplementation on nutrient digestion kinetics was observed in this study, except for improved fecal fat digestibility^57^.

When looking at the trend of availability of the other minerals (Ca, Cu, Mg, Fe, Mn, Zn) and protein digestibility along the GIT (Table S1), a clear drop in availability/digestibility is observed in the proximal gut causing negative ADC values. The drop in ADC is most likely the consequence of the endogenous secretion of minerals to maintain homeostasis and the secretion of, for instance, enzymes like chymotrypsin (from the pancreas) and components containing N, like bile acids, which in fish is generally conjugated with taurine^58,59^. After the proximal intestine, the drop in availability/digestibility was quickly compensated.

In the current study there was a trend for a lowered Ca availability only in the proximal intestine with dietary probiotic supplementation (Fig. 3e). This might relate to increased VFA concentration with probiotic supplementation (Fig. 1b). Bicarbonate secretion (HCO^3-^) in the proximal intestine is required to quickly neutralize the low pH of the stomach, which enters the proximal intestine^53,60^. The higher VFA levels in the proximate intestine associated with probiotic supplementation might lead to an increased secretion of bicarbonate to increase the pH to normal levels. The secretion of bicarbonate is under control of multiple cellular signalling pathways in which Ca^2+^ plays an major role^61^. Therefore, higher secretion of bicarbonate is expected to be linked with a higher influx of Ca into the proximal intestine, which might be the reason for the observed trend for a reduced Ca availability in the proximal intestine in fish fed probiotics supplemented diets. Ca plays an important role in the digestion of lipids. Ca is known precipitate accumulated free fatty acids, thereby Ca can enhance the accessibility of lipase to the emulsified lipids, leading to an increased lipase activity^62,63^. In this study, probiotics resulted in a higher fat digestibility (data shown in^57^), which may be linked to this theorem.

### Microbial community composition and co-occurrence networks

The beneficial effects of *B. amyloliquefaciens* as a probiotic on Nile tilapia have been evaluated in several studies aiming on fish growth performance and immune response^28–30,64–66^. However, the effect of the probiotic *B. amyloliquefaciens* on the gut microbiota composition still remains unclear. Moreover, several studies have evaluated the addition of exogenous enzymes on the gut microbiota of Nile tilapia, turbot and grass carp, showing alterations in the microbial communities, and in some cases even an increase in microbial diversity and richness^67–69^. The combination of enzymes and probiotics in the diets can have complimentary effect by increasing availability of substrates for the probiotic and promoting the abundance of beneficial bacteria, while increasing fibre degradation. In this study, we evaluated the effects of enzyme and probiotic supplementation in the distal gut microbial communities. No significant impact was observed on the richness and diversity between the dietary treatments, potentially due to the large variations among the individuals (Fig. 2). In a study with Nile tilapia, exogenous enzymes (containing phytase, protease and xylanase) and probiotics (containing *Bacillus subtilis, Bacillus licheniformis* and *Bacillus pumilus*) supplementation mildly altered the diversity of the microbial community in the fecal matter^27^. Interestingly, the authors showed a small increase in *Bacillus* species abundance in the gut with probiotic supplementation. In our study, we observed a higher abundance of *Bacillus* species in the groups fed with enzyme and probiotic supplementation (Supplementary Figure S2), showing a potential positive impact on GIT health. Indeed, our VFA and lactic acid analysis indicated a higher microbial activity when probiotics were added. Although this was observed only in the proximal intestine and cannot be directly connected to microbial composition changes, such results indicate potential benefits of probiotic and enzyme supplementation for gut health.

The microbial food webs in the GIT are built largely from the nutrients their host consumes, which act as one of the most important factors in shaping composition and metabolism of the intestinal microbiome^70^. Our analysis showed that the dominant microbial species detected in the distal gut of Nile tilapia were related with protein and carbohydrates metabolism, which could be explained by the high levels of plant materials, including NSP, in the diet. *C. somerae* is an anaerobic microbe which is capable of producing vitamin B12 in fish intestine^71^ and is related to fermentative metabolism of peptides and amino acids^72^. This species has been also reported to produce antimicrobial peptides, that allow it to eliminate other microbes and to occupy most niches in the fish GIT^73^. Our study confirms *C. somerae* to be one of the dominant microbial species in Nile tilapia GIT^74^, potentially due to highly available substrate in the diet and high competitive potential of this taxon. *Brevinema andersonii* was found to be the second dominant species in the Nile tilapia GIT in our study. This species has not been reported before as a dominant species in the Nile tilapia GIT; however, it was previously reported as a dominant species in the GIT of Atlantic salmon (*Salmo salar*), when fed with diets supplied with alginate oligosaccharide, and gilthead seabream, when fed camelina oil^75^. This species was reported to carry genes that are necessary for butyrate production^76,77^, although it was not associated with fatty acid synthase when PICRUSt analysis was applied in the seabream study^75^. Interestingly, a trend for an increase in abundance of *B.andersonii* was found with the addition of probiotics in our study (although not significant; Fig. 5c); such a trend for increase was also observed in the butyric acid content in the proximal gut in fish fed with probiotics (Table 1), implying that probiotics may stimulate the abundance of this species as well as the butyric acid production in the gut. *Romboutsia sedimentorum* and *Bacteroides stercoris* are both obligatory anaerobic microbes^78^, with a broad range of metabolic capabilities with respect to carbohydrate utilization and anaerobic respiration^79^. In our study, both microbial species increased in abundance with enzyme supplementation (Fig. 5c), potentially explained by an increased in carbohydrate substrate. *Paludibacter propionicigenes* is a strictly anaerobic, propionate-producing microbe^80^. From our study, proprionic acid was positively correlated with microbial composition and increased in the distal gut with enzyme supplementation; that could be explained by the increase in the abundance of this species.

Microbial co-occurrence network analysis can be a powerful tool to explore the forces that affect microbial community structure and its dynamics. Such networks have been recently reported in fish to reveal diet-associated shifts^81^, antibiotic effects^82,83^ or understand microbiota dynamics^84^. In our study, probiotics supplementation (CON-PRO and ENZ-PRO) increased the species interactions in the distal gut microbiota when compared with the control treatment (CON-CON). A previous study showed that probiotics enhanced the species-species interaction network in the hindgut of sea cucumber, by increasing the network complexity (clustering co-efficiency and density), which was hypothesized to benefit the intestinal microbiota homeostasis^25^. Besides, we also found that probiotics supplementation enhanced the growth of tilapia with a smaller fraction of dietary energy going to maintenance^57^. A stable intestinal microbiota might be beneficial to the fish with high NSP diet inferences, thus reducing the energy requirement for maintenance. Interestingly, we found that the ratio of positive to negative interactions (co-occurrence patterns) decreased with the addition of the enzyme and probiotics. That could be explained by the breaking down of more complex polysaccharides in the diets, which can serve as a food source for specific microbes^85^. More complex carbohydrates as substrates create different trophic levels in the GIT (different microbes are able to utilize different substrates) thus can potentially support higher diversity^86^. By partly digesting some of those sources, such trophic levels might have been disrupted, creating an environment with high availability and less complexity of substrates in which more microbes have to compete for the same substrates. In the case of the probiotic presence, a higher production of VFAs by the microbial communities may lead to lower pH, thus negatively affecting certain taxa that are less tolerant to such conditions. This agrees with the higher abundance of lactic acid bacteria (*Lactobacillales*) with probiotic and enzyme supplementation (Supplementary Figure S1), leading to higher lactic acid production and a lower pH. Looking at the network ENZ-PRO, the negative interactions mainly originated from species of the order *Clostridiales*, which are known to consist of many fermentative species, producing VFAs^87^. In the network CON-PRO, the negative interactions mainly originated from *B.andersonii*, which is a butyrate-producing bacteria^76^, and was found to increase when probiotics were added, thus potentially explaining the negative impact on other microbial species. Therefore, dietary supplementation of probiotics and enzymes can modify the structure of the microbial communities in the Nile tilapia GIT by altering carbohydrate substrates and VFA production, while enhancing microbial interactions and thus increasing microbiome stability and GIT health.

### Conclusions

To summarize, probiotics supplementation enhanced the total VFA concentration in the proximal GIT. Apart from this increase, the total VFA concentration along the GIT remained rather stable. The contribution of acetic acid to the total concentration of VFA was high with 86–88%, whereby the composition of VFA was not influenced by the dietary treatment, nor did it alter along the GIT. The results on the digestion kinetics suggest that the enzymes were mainly active in the first part of the GIT, with a strong effect on the P availability, which is likely to be linked with a low pH. In line with a higher P availability (and thus expected lower levels of phytate), the availability of calcium, iron, zinc and the apparent digestibility coefficient of the ash fraction was higher with enzyme supplementation. Enzymes and probiotics supplementation did not affect gut microbial composition in the distal gut. The microbial community was largely dominated by five species, related to carbohydrate fermentation and VFA production, mainly propionic acid content. Interestingly, an increase in *Bacillus* and lactic acid microbial species was observed with enzyme and probiotic supplementation, highlighting potential beneficial effects for GIT health. This was also supported by the species to species co-occurrence patterns and network complexity, suggesting that probiotics and enzyme supplementation contribute to a more stable GIT microbiome environment.

## Material and methods

The experiment was approved by the Central Animal Committee (CCD) of The Netherlands under DEC. No. 2018.W-0010.002 and the Ethical Committee judging Animal Experiments of Wageningen University, The Netherlands, and carried out according to Dutch law (Act on Animal Experiments). The study was carried out in compliance with the ARRIVE guidelines (https://arriveguidelines.org/arrive-guidelines).

### Experimental design and animal husbandry

The effect of the dietary supplementation of enzymes and probiotics was tested according to a 2 × 2 factorial arrangement, with 4 replicates per treatment. The first factor compared supplementation versus no supplementation with enzymes, using an enzyme cocktail consisting of phytase (Axtra PHY, *Buttiauxella sp.* phytase at 1000 FTU/kg, DuPont Animal Nutrition) and xylanase (Danisco Xylanase at 6000 U/kg, DuPont Animal Nutrition). The second factor compared supplementation versus no supplementation with probiotic mix (Enviva PRO 202 GT, three strains of *B. amyloliquefaciens* at 60 mg/kg feed). This resulted in: a control diet without enzyme and probiotics (CON-CON), a diet with only the probiotic mix (CON-PRO), a diet with only the enzyme cocktail (ENZ-CON), and a diet with the enzyme cocktail and the probiotic mix (ENZ-PRO). Extruded diets (3 mm pellets) were produced by SPAROS Lda. (Portugal). The basal diet was free of fish meal and formulated with current commonly applied low quality ingredients, rich in dietary NSP (see^57^). Two batches of feed were extruded, with and without the probiotic mix, using heat resistant spores (powder form). Diets with (PRO-CON and PRO-ENZ) and without (CON-CON and CON-ENZ) probiotics had an average measured colony-forming unit (CFU) counts of 7.9 × 10^4^ and 3.0 × 10^3^, respectively. After extrusion, the diets were dried in a vibrating fluid bed dryer. Oils and enzymes in liquid form were coated together onto the pellets under vacuum at the research facilities of the Animal Science Group, Wageningen UR, The Netherlands. The coated diets were refrigerated (4 °C) throughout the experiment.

This experiment consisted of two periods: the first period was a balance period (day 0–42) to determine the faecal nutrient digestibility, nutrient balances and fish growth performance (discussed in^57^). The manuscript by Maas et al.^57^ provides more details about the methodology for the growth trial/balance period. During the second period (day 43–47) digesta and mucus were collected along the gastrointestinal tract to determine the VFA content and kinetics of digestion, as well as the gut microbiome composition, respectively (discussed in present paper). The experiment was performed at the Aquaculture Research Facility (ARF) of the Wageningen University, The Netherlands. Male Nile tilapia (*Oreochromis niloticus*; from the strain Silver NMT™) were obtained from a commercial fish breeder (Til-Aqua international, Someren, The Netherlands). The fish were fed daily 16 g feed kg^-0.8^ d^-1^ which corresponds to approximately 80% of expected satiation feeding, to assure all the feed was eaten in all tanks. The daily feed ration was divided into two equal portions fed at 9:00 and 15:30 h. During the experiment, 16 rectangular glass tanks with an effective volume of 60 L were used. Each tank was stocked with 35 fish with the average body weight of 39 g (± SD 0.47). All tanks were connected to the same common water supply as part of one recirculating aquaculture system (RAS). The RAS water treatment section included a sump, a solid removal unit, UV treatment and a biofilter (trickling filter). Each tank was connected to a swirl separator from AquaOptima AS (internal diameter of 24.5 cm and 44 cm column height) to collect faeces. Each swirl separator had a detachable glass bottle to collect faeces and uneaten pellets connected at the bottom outlet. Water flow per tank was maintained at 7 L/min during the experiment. Each tank contained an air stone. The photoperiod was 12 h light: 12 h dark, switching the light on at 7.00 am. Water quality parameters were monitored three times a week, before the first feeding. The water temperature was maintained at 27.5 °C (± 0.2) and pH ranged between 7.0 and 7.9 (mean 7.44 ± SD 0.29). DO level in water inlet to the solid removal unit (common outflow of tanks) in the RAS never dropped below 5.4 mg/L (mean 6.27 ± SD 0.46). At stocking, the conductivity was 5000 µS/cm, and was gradually declined to 4000–3000 µS/cm at the end of the first week. Total ammonia nitrogen, nitrite-N and nitrate–N during the experiment, remained below than 0.25, 0.15 and 500 mg/L, respectively.

### Sampling procedure

Samples for body composition and gut microbiota were taken at the end of the balance period (period 1), these fish were 24 h deprived of feed prior to sampling. All other samples were taken at the end of period 2 on fed fish.

For gut microbiota, 3 fish per tank were randomly selected, euthanized with an overdose of 2-phenoxyethanol (3 ml/L) and weighted. Fish were disinfected with 70% ethanol before dissection. The distal gut was separated from the rest of the gut and divided into two by length, and the first 5 cm in the direction of the anus was sampled. The gut was gently squeezed to ensure any remaining digesta were removed. The bench surface and dissection tools were disinfected with 70% ethanol and sterile water. The collected gut samples were transferred to cryotubes and submerged in liquid nitrogen before storing at − 80 °C until further analysis for gut microbiome.

Fish not taken for body composition and microbiota at the end of the balance period, were continued to be fed for 3 or 4 days using approximately a 10% higher feeding ration as the balance period using a belt feeder (feeding 24 h). This was done in order to ensure that fish were full so that digesta could be collected along the gastrointestinal tract (GIT). On day 46 and 47, eight tanks (2 replicate per treatments per sampling day) were sampled and all fish were euthanized by an over-dosed of 2-phenoxyethanol (3 ml/L) for digesta collection. In each tank, feeding was stopped approximately 1 h before sampling the fish, to prevent collection whole pellets in the stomach. Samples of digesta were collected in four sections of the GIT: stomach and proximal, middle, and distal part of the intestine. The proximal part was taken from the stomach (after the pyloric part of the stomach) until the spiral part of the intestine (gut becomes thinner), the division of the middle and distal part was done based on having equal lengths per section. Digesta per section was pooled per tank and collected in pre-weighted crucibles. Crucibles and fish not directly sampled were kept on ice at all time to stop bacterial activity and prevent degradation of the faeces. Digesta was collected for dry matter, crude protein, ash, P, Ca, Cu, Mg, Mn, Fe, Zn and VFA measurements. VFAs were measured in the digesta as an indication for fermentation in the GIT. Freshly collected digesta (0.5 mL) was added to 0.5 mL of buffer (1 mL distilled water and 50 µL phosporic acid) with *iso*-caproic acid as internal standard in cryotubes and stored at -20 °C until analysis. For the analyses of lactic acid, 1 mL of digesta (in duplicate) was collected and stored (no buffer) in cryotubes at − 20 °C. The crucibles with the remaining digesta were weighted to determine the DM content of the digesta in the different segments of the GIT and dried at 70 °C until further analysis.

### VFA and lactic acid content along the gastrointestinal tract

Samples stored for VFA analyses (− 20 °C) were thawed at room temperature, mixed (vortex mixer) and centrifuged for 10 min at 10.000 rpm. Supernatant was taken and put in 2 mL clear glass vails with insert and sealed using aluminum caps with silicone septa. The concentrations of acetic, propionic, *iso*-butyric, butyric, *iso*-valeric and valeric acid were measured as previously described^88^; VFA were separated by gas chromatography using a HP-FFAP (30 m × 0.32 mm, 0.25 µm) column from Agilent (Santa Clara, California, USA) and hydrogen as the mobile phase with detection by flame ionization detector. Quantification of VFA was based on a chemical standard solution (Merck, Hohenbrunn, Germany) after internal standard correction. VFA concentrations were expressed in mmol per L of fresh digesta.

For the lactate content measurement, digesta samples stored at − 20 °C were thawed at room temperature. Prior to analysis, digesta were centrifuged for 30 min. at 14,000 × g at 4 °C. Subsequently, supernatant was transferred to Amicon 10 K spin columns (Z677108-96EA, Sigma Aldrich). Spin columns were centrifuged at 14,000 × g for 20 min. and filtrate was collected. Filtrate was used for lactate analysis using the Lactate Colorimetric Assay Kit II (K627, Biovision) according to the manufacturer’s instructions. Briefly, filtrate was tested at three different dilutions; 5, 10 and 50 times diluted in “Lactate Assay Buffer” and 50 µL sample or diluted sample was transferred to a 96-wells plate. Subsequently, 50 µL reaction mix composed of Lactate Substrate Mix (2 µL), Lactate Enzyme Mix (2 µL), and Lactate Assay Buffer (46 µL), was added to each well and incubated for 30 min at room temperature. Optical density was measured at 450 nm and concentrations of extracellular lactate were calculated based on a lactate calibration curve supplied in the kit.

### Digesta nutrient composition analysis and digestibility calculation

The digesta were ground using a lab jar mill (stainless) prior to the analysis, feed was analyzed as whole pellets^89^. Collected digesta and feed were analyzed gravimetrically for dry matter (DM) by drying at 103 °C for 4 h until constant weight. Following the DM determination, ash content was determined gravimetrically by incineration in a muffle furnace for 4 h at 550 °C (ISO 5984, 1978). Ashed samples were transferred to volumetric flasks and dissolved in concentrated sulphuric acid solution by autoclaving. Samples were subsequently diluted in water and filtered using a syringe filter (45 µm pores). Finally, Yttrium (Y), Phosphorous (P), calcium (Ca), magnesium (Mg), manganese (Mn), iron (Fe) and zinc (Zn) were analyzed using inductively coupled plasma-mass spectrometry (ICP-OES) according to the standard NEN 15,510 (2007). The total nitrogen content was measured in feed using the Kjeldahl-method (ISO 5983, 1997) and in digesta according to Dumas method^90^, calculating crude protein as N × 6.25 (protein conversion factor).

The apparent digestibility coefficient (ADC) of mineral and crude protein was calculated as ADC (%) = 100 × [1 − (Y_i_ × amount nutrient in digesta)/ (Y_f_ × amount nutrient in feed)], where Y_i_ (g/kg dry matter) is the concentration of Y in the feed and Y_f_ (g/kg dry matter) is the concentration of Y in the digesta from the four sections along the GIT.

### DNA extraction and sequencing

The distal gut samples were sent to BaseClear (Leiden, the Netherlands) for DNA extraction using a commercial kit (*ZymoBIOMICS DNA Miniprep Kit, Zymo Research—Cat. No D4300)*. Library preparation was performed according to the 16S Metagenomic Sequencing Library Preparation—Preparing 16S Ribosomal RNA Gene Amplicons for the Illumina MiSeq System,. PCR-amplified V3-V4 region of 16S rRNA was sequenced using the Illumina MiSeq 2000 Next Generation system. Amplification of the V3-V4 region was performed under the following conditions: 98 °C for 30 s, followed by 25 cycles of 98 °C for 10 s, 55 °C for 30 s and 72 °C for 30 s, and a final elongation step at 72 °C for 5 min. The PCR product was cleaned using AMPure XP beads (Beckman Coulter) and quantified for the fragments containing the Illumina adaptors. Products were quantified using a standard curve with serial DNA concentrations (0.1–10 nM). Samples were equimolarly diluted to a concentration of 4 nM and prepared for sequencing according to the manufacturer’s instructions.

Paired-end sequence reads were collapsed into so-called *pseudoreads* using sequence overlap with USEARCH (version 9.2)^91^. After chimera removal, classification of these pseudoreads was performed based on the results of alignment with SNAP (version 1.0.23)89 against the RDP database (release 2.11) for bacterial organisms^92^. Sequencing data can be found at the NCBI (SRA) database under the study accession code SRP307674.

### Microbial community analysis

In total, 48 tilapia gut samples were sequenced, giving a total of 2,200,267 reads, after quality filtering. Per sample, the sequencing depth ranged between between 6300 and 78,043. Four samples (1 from CON-CON and 3 from ENZ-CON) were removed from analysis due to the low sequencing depth (< 27,000 which was the next highest sequencing depth). The sequencing data of the remaining 44 samples were rarified at the threshold of 27,000 reads sequencing depth. Alpha-diversity was assessed using Shannon diversity index and observed richness (the number of Operational taxonomic units, OTUs) for each sample. Beta-diversity was assessed using the Bray–Curtis distance metric, and clustering analysis was performed by Principle Coordinate Analysis (PCoA), using the Primer software (Version 6).

Network construction was performed using CoNet^93^ between the different treatment groups, to assess the microbial co-occurrence relationships between microbes, using the recommended parameters. For the network analysis, the following parameters were evaluated: A. Clustering coefficient, which is the ratio between existing and possible connections between a node’s neighbours—it is measuring the degree to which several nodes in a network cluster together; B. Network density, which is defined as the ratio of the number of total edges to the number of possible edges between all the nodes of the network; C. Network heterogeneity, which is an index that quantifies the diversity of connections between nodes in networks, even with different topologies—it ranges from 0 to 1, with 1 referring to maximum heterogeneity, i.e. when each one of the nodes is connected to all the other nodes. This measure may indicate the stability and robustness of a network with respect to perturbations from various external factors. D. Positive to negative edges ratio, indicating co-presence versus mutual exclusion patterns.

### Statistical analysis

The effect of enzyme and probiotic supplementation and their interaction on VFA and lactate concentration, minerals absorption and protein digestibility were tested by two-way ANOVA using general linear model in SPSS software (IBM, version 25), when normality and equality of variance were confirmed; otherwise rank-transformation was applied. The significance of individual treatment was compared using Tukey HSD when the effect was significant (*P* < 0.05).

The alpha-diversity scores were compared among the four treatments by nonparametric t-test. The effects of enzymes and probiotics and their interactions on microbial composition were analyzed by two-way PERMANOVA using Primer (Version 6). Moreover, Pearson correlations between the OTU matrix (resembled by Bray–Curtis distance) and the host performance predictors (fish body weight—BW, digestibility and VFA content in the distal gut) were tested by distance based linear modeling (DistLM in Primer Version 6) and the results were visualized in a PCoA diagram. The similarity percentage among the four treatments and the contributions from each species were calculated by similarity percentage (SIMPER) analysis using PAST software (Version 4). For the microbial interactions, significance of the tested parameters was assessed by rarefying the table, using subsampling at 19,000 reads sequence depth (70% of the initial reads), and performing the analysis for each group using the Mann–Whitney test.

## Supplementary Information

Supplementary Information.
