# Supplementary material for: Exogenous enzymes and probiotics alter digestion kinetics, volatile fatty acid content and microbial interactions in the gut of Nile tilapia
Source: Sci Rep. 2021 Apr 15;11:8221. doi: 10.1038/s41598-021-87408-3 (PMC8050056; doi:10.1038/s41598-021-87408-3)
Supplement: Supplementary file 1 — Supplementary Information. [file 41598_2021_87408_MOESM1_ESM.pdf]

Supplementary materials

Exogenous enzymes and probiotics alter digestion kinetics, volatile fatty acid content and microbial interactions in the gut of Nile tilapia

Roel M. Maas[a1], Yale Deng[a1], Yueming Dersjant-Li[b] , Jules Petit[a], Marc C.J. Verdegem[a], Johan W. Schrama[a], Fotini Kokou[a*]

[a]*Aquaculture and Fisheries Group, Wageningen University and Research, Wageningen, The Netherlands*

[b]*Danisco Animal Nutrition, Oegstgeest, The Netherlands*

Table S1. The apparent digestibility coefficient (ADC, %) along the gastrointestinal tract of Nile tilapia.

Enzymes	CON		ENZ		SEM	*P* values		
Probiotics	CON	PRO	CON	PRO		ENZ	PRO	ENZ*PRO
ADC (%)								
**Stomach**								
Dry matter	-0.4	-0.5	2.9	0.2	0.9	ns	ns	ns
Crude protein	0.1	6.9	4.0	7.8	1.8	ns	*	ns
Ash	21.8	20.1	26.7	20.9	1.3	ns	ns	ns
Phosphorous	13.0	16.9	17.8	13.9	0.9	ns	ns	#
Calcium	18.2	19.8	20.5	13.4	1.1	ns	ns	*
Copper	3.3	9.2	9.3	0.8	1.7	ns	ns	#
Magnesium	20.3	11.2	25.3	18.7	3.7	ns	ns	ns
Iron	-20.3	-31.8	-29.9	-26.0	0.9	ns	ns	ns
Manganese	9.0	9.6	8.7	-3.4	4.8	ns	ns	ns
Zinc	2.9	13.7	-1.4	-6.4	2.1	**	ns	*
**Proximal**								
Dry matter	-53.0	-85.8	-37.7	-46.0	6.1	*	ns	ns
Crude protein	-43.8	-68.3	-20.1	-22.2	2.3	*	ns	ns
Ash	-95.7	-140.2	-74.5	-77.3	8.0	**	ns	ns
Phosphorous	2.2	-4.1	23.7	22.5	3.0	**	ns	ns
Calcium	-12.2	-18.9	-10.4	-15.8	1.7	ns	#	ns
Copper	-137.9	-216.4	-160.2	-171.6	15.9	ns	ns	ns
Magnesium	-65.2	-119.2	-77.0	-88.0	10.3	ns	ns	ns
Iron	-84.1	-178.2	-98.0	-101.9	6.4	ns	ns	ns
Manganese	-19.8	-36.6	-31.5	-44.7	17.4	*	**	ns
Zinc	-48.1	-51.9	-59.1	-67.3	2.1	*	ns	ns
**Middle**								
Dry matter	33.8	28.7	34.6	36.2	1.1	#	ns	ns
Crude protein	57.0	55.6	59.3	62.3	0.9	*	ns	ns
Ash	-20.0	-27.0	-14.8	-10.9	1.7	*	ns	ns
Phosphorous	27.8	27.5	41.8	44.6	1.5	***	ns	ns
Calcium	7.3	10.6	15.7	16.2	1.7	#	ns	ns
Copper	2.2	2.9	-5.8	-1.4	2.5	ns	ns	ns
Magnesium	12.5	9.4	13.7	17.0	1.5	ns	ns	ns
Iron	-22.9	-41.0	-36.0	-56.9	1.0	ns	*	ns
Manganese	-5.5	-23.2	-11.0	-22.6	4.3	ns	**	ns
Zinc	-14.2	-14.5	-20.1	-22.5	1.8	**	ns	ns
**Distal**								
Dry matter	45.7	47.0	46.5	49.2	1.6	ns	ns	ns
Crude protein	69.7	70.4	69.7	72.7	1.5	ns	ns	ns
Ash	-6.8	-6.4	3.6	6.5	2.1	*	ns	ns
Phosphorous	29.2	28.9	42.0	46.8	1.2	***	ns	ns
Calcium	7.1	9.3	16.8	20.4	1.6	**	ns	ns
Copper	27.7	30.2	15.8	17.6	4.0	ns	ns	ns
Magnesium	21.9	25.2	27.0	35.9	2.8	ns	ns	ns
Iron	-34.1	-26.2	-36.8	-59.4	1.3	*	ns	#
Manganese	-6.0	-22.1	-9.2	-24.8	4.0	ns	***	ns

Zinc	-10.3	-7.0	-13.5	-19.2	1.6	*	ns	ns

Note: CON, no supplementation; ENZ, enzyme (effect) supplementation; PRO, probiotic (effect) supplementation; ENZ*PRO, interaction effect ns, not significant, # $P < 0.1$, * $P < 0.05$, ** $P < 0.01$, *** $P < 0.001$.

!

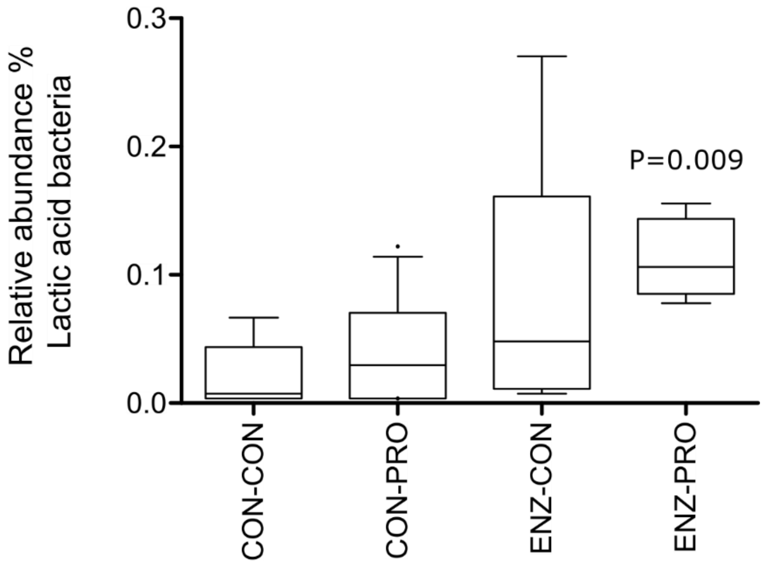

Supplementary Figure S1. Relative abundance of lactic acid bacteria in the Nile tilapia distal gut. Significance from CON-CON is indicated after Mann-Whitney test. CON-CON, no enzymes or probiotics added; CON-PRO, probiotics added; ENZ-CON, enzymes added; ENZ-PRO, enzymes and probiotics added.

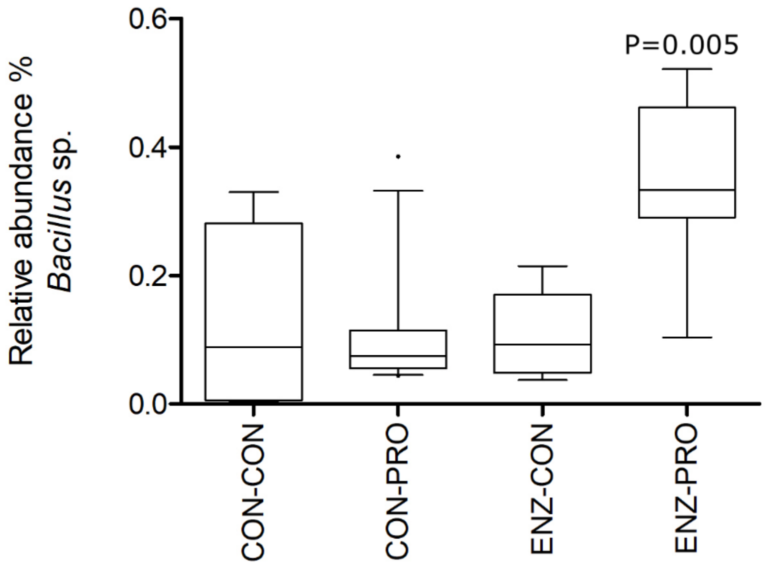

Supplementary Figure S2. Relative abundance of Bacillus species in the Nile tilapia distal gut. Significance from CON-CON is indicated after Mann-Whitney test. CON-CON, no enzymes or probiotics added; CON-PRO, probiotics added; ENZ-CON, enzymes added; ENZ-PRO, enzymes and probiotics added.
